# The prediction of round window visibility at posterior-tympanotomy for cochlear implantation with “black bone” magnetic resonance imaging: Author lists

**DOI:** 10.1007/s00405-025-09718-w

**Published:** 2025-10-01

**Authors:** Hoi Ming Kwok, Cameron Spence, Emily Hocknell, Irumee Pai, Steve Connor

**Affiliations:** 1 Department of Diagnostic and Interventional Radiology, Princess Margaret Hospital Kowloon West Cluster, Kowloon, Hong Kong SAR China; 2 Department of Imaging and Interventional Radiology, Chinese University of Hong Kong, Sha Tin, Hong Kong SAR China; 3 Department of Radiology, University Hospitals Dorset, Bournemouth, BH7 7DW UK; 4 Department of Ear, Nose and Throat Surgery, Guy’s and St Thomas’ Hospital, London, SE1 9RT UK; 5 School of Biomedical Engineering and Imaging Sciences, King’s College London, London, SE1 7EH UK; 6 Department of Neuroradiology, King’s College Hospital, London, SE5 9RS UK; 7 Department of Radiology, Guy’s Hospital and St Thomas’ Hospital, London, SE1 9RT UK

**Keywords:** Black bone magnetic resonance imaging, Round window, Cochlear implantation, Otology, Pre-operative imaging

## Abstract

**Purpose:**

Although computed tomography (CT) can predict round window (RW) visibility at posterior tympanotomy pre-operatively, there is a trend towards the application of using magnetic resonance imaging (MRI) alone for cochlear implant (CI) planning. This study assessed the potential of a novel “Black Bone” (BB) MRI sequence to determine RW visibility during posterior tympanotomy.

**Methods:**

Patients underwent BB MRI as part of pre-operative CI planning. Two independent radiologists performed 5 landmark-based MRI measurements. RW visibility was recorded during posterior tympanotomy as > 50% or < 50% visibility. Patients with undefinable landmarks or absent surgical grading were excluded. Mann-Whitney U or t-tests compared MRI measurements to RW visibility whilst step wise logistic regression determined significant predictors.

**Results:**

86 patients (40 male; median 10.5 years) and 129 implanted ears (43 unilateral, 43 bilateral; 52 adult, 77 paediatric) were evaluated. There were 109/129 ears with > 50% and 20/129 ears with < 50% RW visibility. The external auditory canal (EAC) angle was increased in paediatric patients with > 50% RW visibility (*P* = 0.033; AUROC 0.689; 73.4% sensitive, 69.2% specific with threshold 6.2^o^) with ICC = 0.598. The facial recess distance, facial nerve location, and modified RW niche angle were increased in adult patients with > 50% RW visibility but only modified RW niche angle was significant on stepwise regression (*P* = 0.016; AUROC 0.805; 82.2% sensitive, 71.4% specific with threshold 17.95^o^) with ICC = 0.578.

**Conclusion:**

The visibility of RW at posterior tympanotomy may be determined with pre-operative BB MRI. The optimal predictive measurements differ between adult and paediatric patients and there is superior performance in adults.

## Introduction

Cochlear implantation remains the standard therapy for auditory rehabilitation in patients with congenital or acquired severe-to-profound deafness [1]. Pre-operative imaging with computed tomography (CT) or magnetic resonance imaging (MRI) is primarily performed to assess the patency and morphology of the cochlea as well as the integrity of the retro-cochlear auditory pathways. CT has the advantage of depicting the bony anatomy along the surgical approach and studies have highlighted its ability to predict round window (RW) visibility [1–8]. However, MRI provides superior evaluation of cochlear nerve integrity, labyrinthine patency, and inner ear malformations without ionizing radiation, and there is a trend towards the utilization of MRI alone in pre-surgical planning. In this context, it would be beneficial to develop an MRI technique which was also able to predict surgical access to the RW [8].

A RW insertion is preferred in cochlear implantation since it allows for a more confident scala tympani placement with reduced intracochlear trauma, and improved hearing outcomes [9]. The surgical approach is via a posterior tympanotomy which was first described by Jansen in 1958. This approach involves accessing the facial recess—a triangular space bounded by the mastoid segment of the facial nerve medially, the chorda tympani laterally, and the incudal fossa superiorly [10]. Accurate preoperative prediction of RW visibility at posterior tympanotomy would be of benefit since it would allow pre-surgical preparation for challenging surgical access and the potential requirement for cochleostomy.

Black Bone (BB) MRI, introduced in 2012 [11], has emerged as a radiation-free alternative for osseous temporal bone imaging [12]. Initially applied in paediatric craniofacial and spinal disorders [13–20], BB-MRI demonstrates excellent visualization of key surgical landmarks, including the mastoid facial nerve (100%) and chorda tympani (72%) [12] but the ability of BB-MRI to evaluate surgical access for CI has not been evaluated.

The objective of this study was to determine the ability of BB-MRI to predict RW visualization at surgery using anatomical landmark-based measurements in adult and paediatric CI patients.

## Materials and methods

### Patient inclusion and exclusion

**Fig. 1:**
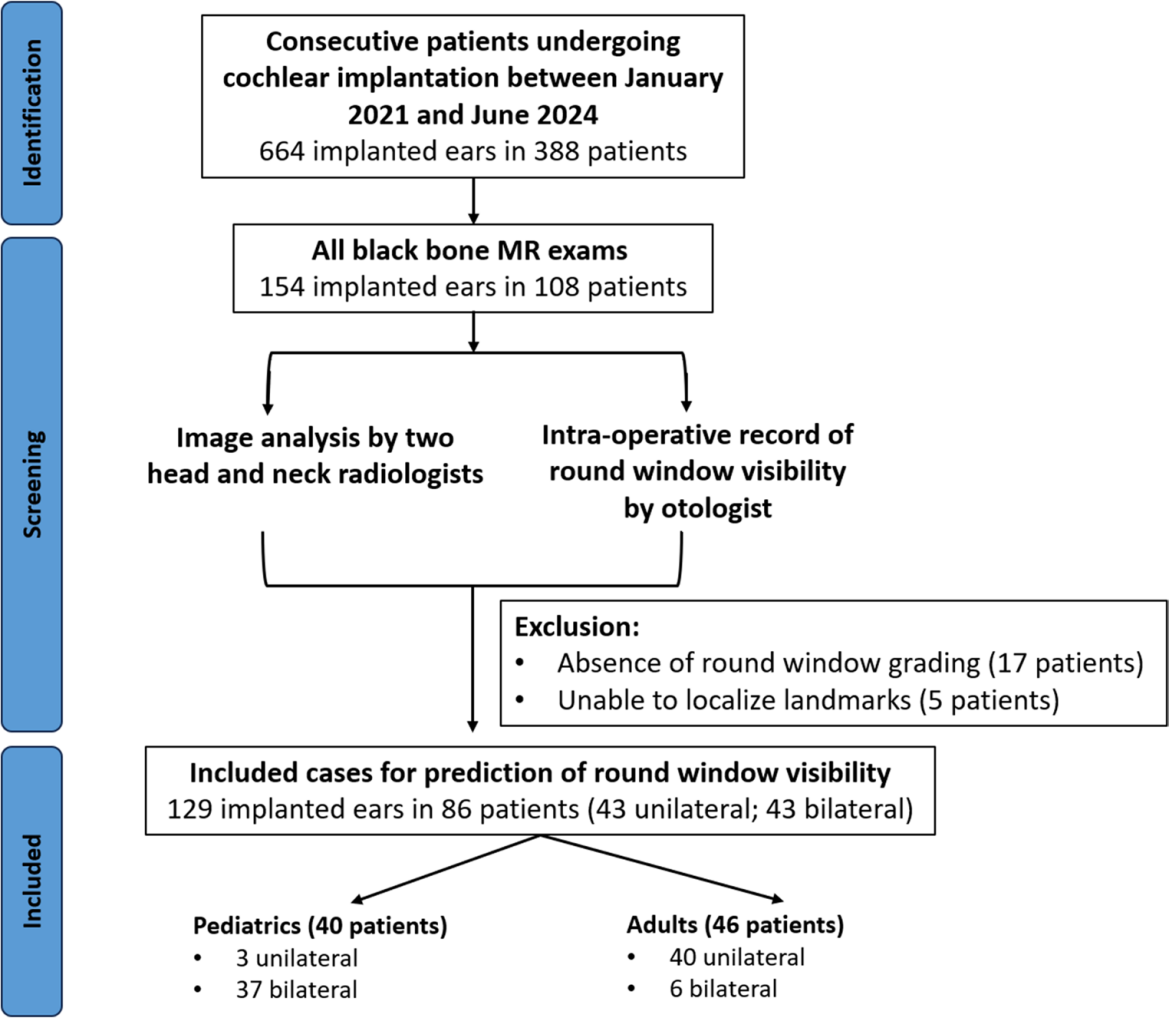
Flowchart showing the inclusion and exclusion of cases

This study underwent local institutional review by the Clinical Research Analytics Governance group (CRAG) at Guy’s and St. Thomas’ NHS Foundation Trust and was approved, including waived informed consent (GSTT Electronic Record Research Interface, IRAS ID: 257283, Rec Reference: 20/EM/0112). This was a retrospective cross-sectional study from a single institution. Consecutive patients undergoing cochlear implantation at GSTT between January 2021 and June 2024 were identified from the surgical database. These were cross-referenced to the PACS system (Sectra AB, Sweden) to identify those who had undergone preoperative BB-MRI. Patients were excluded if the surgical grading of RW visibility score had not been recorded or if it was not possible to identify all BB-MRI-based anatomical landmarks to derive the measurements (Fig. 1).

### MRI protocols and techniques

Axial BB MRI images of the temporal bone were performed on the same 1.5 Tesla system (Magnetom Aera; Siemens AG, Germany) using a 20-channel head and neck coil (Table 1).

**Table 1:** Sequence parameters of the “black bone” MRI

	Black Bone sequence
Scanner	1.5 Tesla system (Magnetom Aera; Siemens AG, Germany)
Coils	20-channel head and neck coil
Sequence type	Volumetric SPGR BB MRI
TR/TE/FA	11ms/5.07ms/5^o^
FOV	224 × 168 × 144 mm^3^
Voxel size	Acquired 0.7 × 0.7 × 1 mm^3^ and interpolated to 0.4 × 0.4 × 1.0mm^3^
No. of averages	4
Total acquisition	GRAPPA = 2 4.29 min

*BB MRI* black bone MRI, *SPGR* spoiled gradient echo, *GRAPPA *545 generalized autocalibrating partial parallel acquisition

### Selection and definition of anatomical MR-based landmarks and measurements

A literature review was carried out following a Boolean search using the PubMed database in July 2024, using the keywords “computed tomography” AND “round window,” AND/OR “posterior tympanotomy.” Previous CT measurements [2–6, 21] were collated and adapted according to the anatomical landmarks clearly identifiable on the BB MRI sequence. The detailed definitions for the measurements are summarized (Table 2).

**Table 2:** Definitions of different radiological measurement on BB-MRI

EAC line	From the bony-cartilaginous junction to the tympanic annulus along the bony-mucosal surfaces of the posterior wall of the EAC on an axial Sects. [3,4,5]. If the line could not be drawn, it was created at the nearest MR slice and then moved to the original slice at the same angle.
Basal turn line	Parallel to and centrally within the inferior segment of the cochlear basal turn on an axial Sects. [3,4,5].
Facial recess width (in mm)	The vertical distance between the EAC line and the anterolateral margin of the facial nerve [3,4,5].
Facial recess distance (in mm)	The vertical distance from the coronal plane to the anterior margin of the facial nerve [3]
Facial nerve location (in mm)	The vertical distance between the anterolateral surface of the facial nerve and the basal turn line [3,4]. A positive value was applied when the anterolateral margin of the nerve was posterior to the line while a negative value was applied when the anterolateral margin of the nerve was anterior to the basal turn line.
Modified round window niche angle	The angle between the coronal plane and the line connecting the dorsal cochlear promontory to the anterior margin of the facial nerve [3,4].
EAC angle	The angle between the EAC line and the basal turn line [3].
*EAC* external auditory canal	

**Fig. 2:**
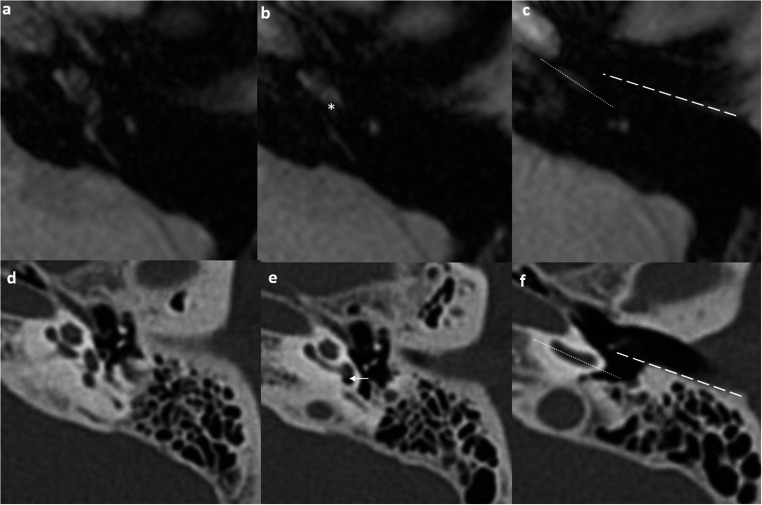
Standard axial MR section from the BB-MRI sequence where the measurements are performed (**A **to** C**). The axial section showing the basal turn of the cochlea in continuity to the vestibule is identified (**A**). The axial MR image one slice below the prior slice shows the dorsal aspect of the promontory of the basal turn of the cochlea (asterisk) as the surrogate for the round window niche for measurement of modified round window niche angle (**B**). The axial MR image two slices below the prior slice in b showing the mid external auditory canal (EAC) level for measurement of the EAC line (dotted line) and the basal turn line (dash line) (**C**). This slice allows the measurements of mastoid facial nerve location. Corresponding axial high-resolution CT showing similar axial sections as BB-MRI (**D to F**). The axial CT image showing the basal turn of the cochlea in continuity to the vestibule (**D**). The axial CT image showing round window niche (arrow) (**E**). The axial CT image at mid EAC level showing the EAC line (dotted line) and basal turn line (dash line) (**F**)

All radiological measurements incorporated two standardized reference lines: the EAC line and basal turn line, enabling both linear and angular measurements (Fig. 2). The EAC line extended from the bony-cartilaginous junction to the tympanic annulus along the bony-mucosal surfaces of the posterior wall of the EAC on an axial Sects. [3–5]. The basal turn line was parallel to and central within the inferior segment of the cochlear basal turn on an axial Sects. [3–5].

**Fig. 3:**
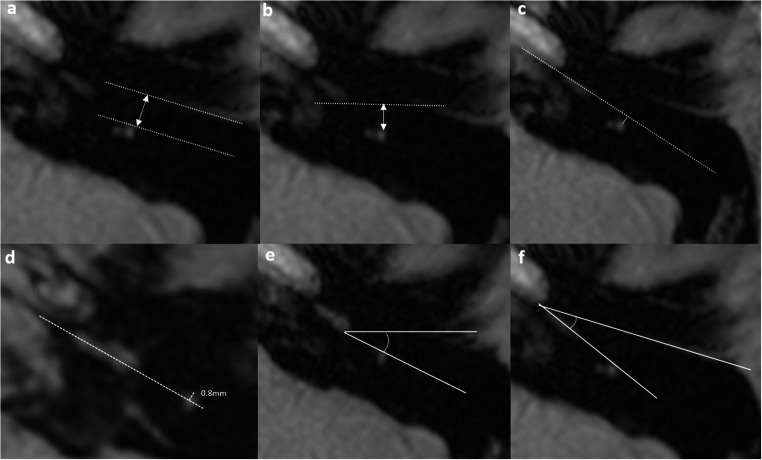
BB-MRI based measurements. The external auditory canal (EAC) line is drawn along the bony-mucosal surfaces of the posterior wall of the EAC on axial slice. The basal turn line is drawn from the center and is parallel to the cochlear basal turn on the axial slice. Facial recess width (mm) is measured by the vertical distance between the external auditory canal line and the anterolateral margin of the facial nerve (**A**). Facial recess distance (mm) is measured by the vertical distance from the coronal plane to the anterior margin of the facial nerve (**B**). Facial nerve location (mm) is the vertical distance between the anterior lateral surface of the facial nerve and the basal turn line (**C**). If the facial nerve location is anterior to the basal turn line, a negative value is recorded i.e. −0.8 mm in this example (**D**). The modified round window niche angle is defined as the angle between the coronal plane and the line connecting the middle part of the round window niche to the anterior margin of the facial nerve. In the current study, we used the dorsal aspect of the cochlear promontory as the surrogate for the round window niche (**E**). The EAC angle is the angle between the EAC line and the basal turn line (**F**)

The mastoid facial nerve was measured at the mid-EAC level, standardized to two slices below the cochlear basal turn reference slice (Fig. 3). Facial recess width (in mm) was measured by the vertical distance between the EAC line and the anterolateral margin of the facial nerve [3–5]. Facial recess distance (in mm) represented the vertical distance from the coronal plane to the anterior margin of the facial nerve [3]. Facial nerve location (in mm) was defined as the vertical distance between the anterior lateral surface of the facial nerve and the basal turn line [3, 4]. Since BB-MRI is unable to define the bony outline of the RW niche, the dorsal aspect of the cochlear promontory was used as a surrogate landmark. The modified round window niche angle was defined as the angle between the coronal plane and the line connecting the dorsal aspect of the cochlear promontory to the anterior margin of the facial nerve [3, 4]. The EAC angle was the angle between the EAC line and the basal turn line [3] (Fig. 3).

#### Image analysis

Two head and neck radiologists (H.M.K., 9 years and C.S., 5 years radiology experience), independently analyzed all BB-MRI studies on a Sectra Workstation IDS7 (Sectra AB, Linköping, Sweden) whilst blinded to surgical RW visibility assessment.

### Surgical grading of RW visibility

The intraoperative evaluation served as the reference standard for RW visibility assessment. RW visibility was scored according to the St. Thomas’ Hospital classification system following posterior tympanotomy [22] as part of standard clinical practice. The grading system describes: Type I (complete RW membrane exposure); Type IIa (> 50% RW membrane visibility); Type IIb (< 50% RW membrane visibility); and Type III (membrane positioned posteriorly adjacent to the facial nerve). The RW grades for each patient were obtained from the surgical records. For analytical purposes, the classification was dichotomized a priori into > 50% visibility (combining Types I and IIa) versus < 50% visibility (Types IIb and III), which was considered a clinically significant threshold as the latter frequently necessitates cochleostomy [22]. The time interval between preoperative imaging and surgical RW grading was recorded.

### Statistical analysis

Statistical analysis was conducted using IBM SPSS version 29.0.2.0, Armonk, NY. The normality of distribution was verified using the Shapiro-Wilk test. Continuous variables that were normally distributed were expressed as mean ± standard deviation (SD) and compared using independent samples t-tests. Continuous variables that were not normally distributed were reported medians with interquartile ranges (IQR) and the Mann-Whitney U test was used for group comparisons. Categorical variables were presented as frequencies and percentages. To identify significant predictors associated with the outcome, forward stepwise logistic regression was performed using a threshold *p*-value of < 0.05 for variable entry. Diagnostic performance of significant predictors was evaluated using receiver operating characteristic (ROC) curve analysis, with the area under the curve (AUC) used to assess predictive accuracy. Optimal cutoff values were determined by maximizing the Youden index (sensitivity + specificity − 1). Interobserver reliability was quantified using the intraclass correlation coefficient (ICC) for continuous measurements, with values interpreted as follows: <0.5 (poor reliability), 0.5–0.75 (moderate reliability), 0.75–0.9 (good reliability) and 0.9 (excellent reliability).

## Results

### Demographic results

Of the initial 108 patients in whom BB MRI was performed for pre-operative CI planning, there were 17 (15.7%) patients excluded due to unavailable RW grading and an additional 5 (4.6%) exclusions due to inability to localize anatomical landmarks on image review (Fig. 1). The final cohort comprised 86 patients (129 implanted ears), comprising 40 males (62 ears) and 46 females (67 ears), with a median age of 10.5 years (IQR 4.5–53.2 years). The cohort consisted of 46.5% paediatric patients (40/86; 3 unilateral, 37 bilateral) and 53.5% adults (46/86; 40 unilateral, 6 bilateral), with near-equal distribution between right (64/129, 49.6%) and left (65/129, 50.4%) implanted ears. The median interval between preoperative imaging and RW visibility grading differed between cohorts, with paediatric cases demonstrating shorter delays (176 days, IQR 111–248) compared to adult cases (237 days, IQR 144–285).

### Comparison of BB-MRI landmark-based measurements with RW visibility (Figure 4)

**Fig. 4:**
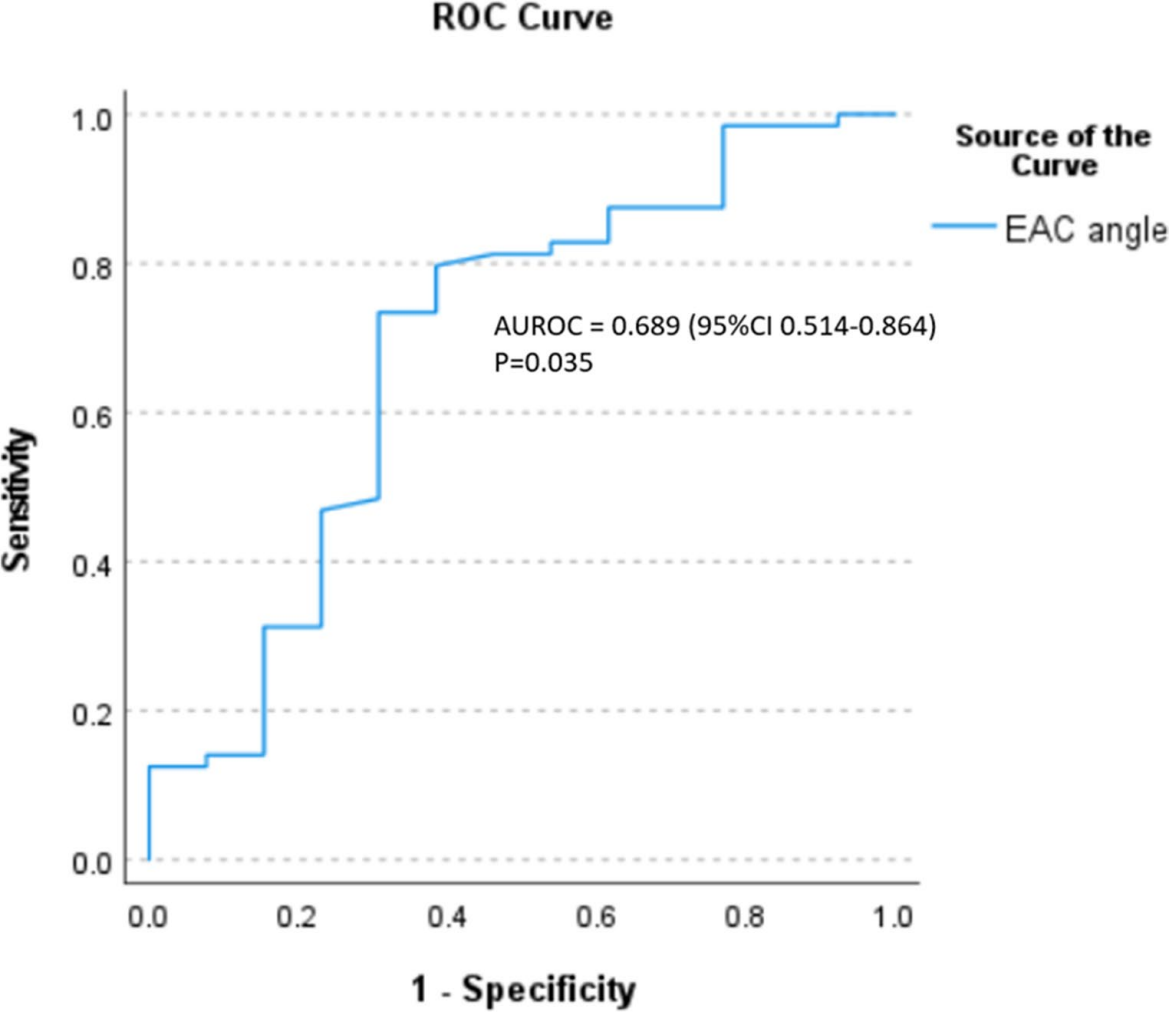
AUROC of external auditory canal (EAC) angle in pediatric patients

**Fig. 5:**
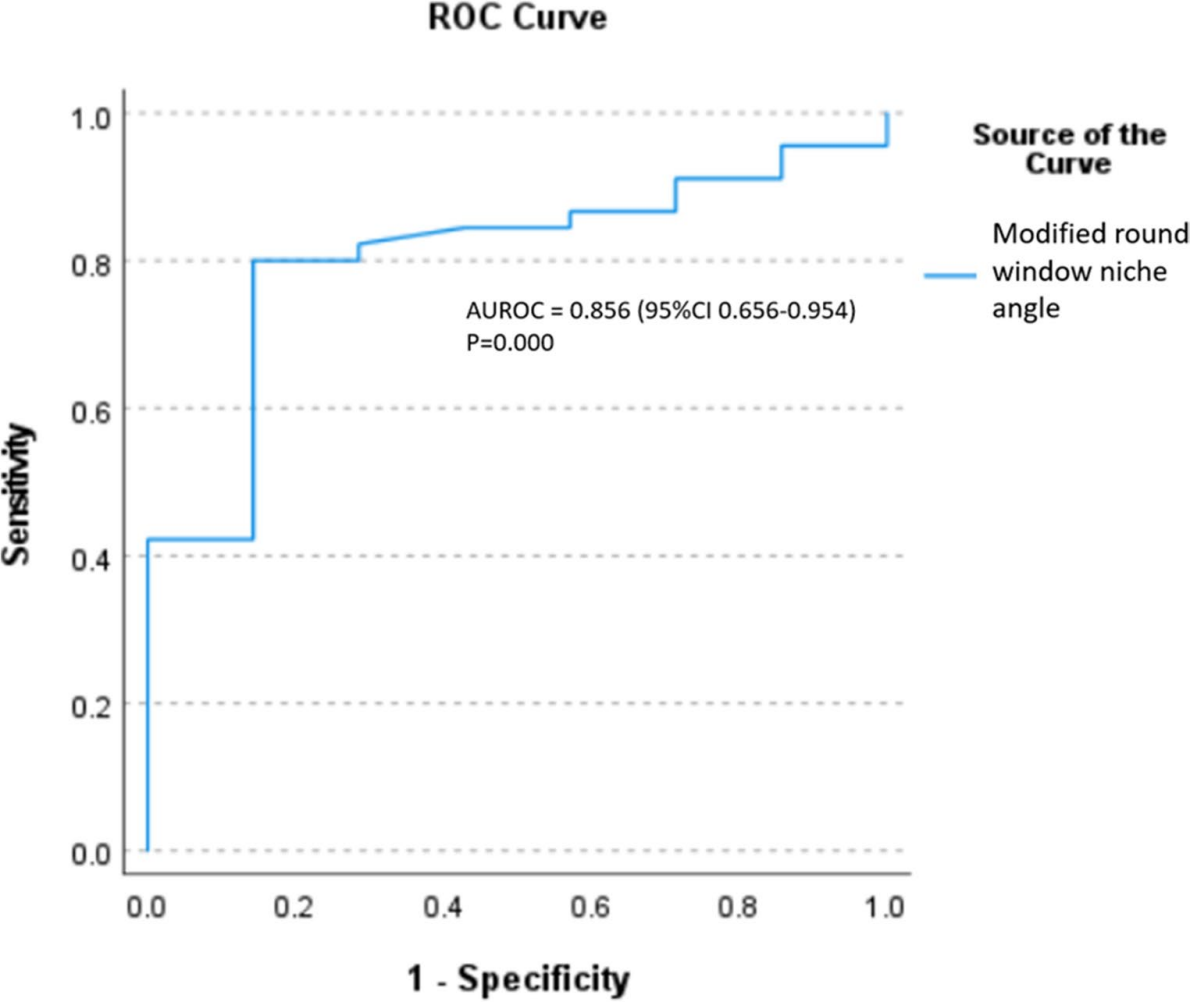
AUROC of modified round window niche angle in adult patients

The majority of implanted ears (109/129, 84.5%) demonstrated > 50% RW visibility (Type I: 74, 57.4%; Type IIa: 35, 27.1%), whilst 20 ears (15.5%) had < 50% visibility (Type IIb: 17, 13.2%; Type III: 3, 2.3%) (Table 3). In paediatric patients, the EAC angle was the only measurement to be significantly associated with RW visibility. It was significantly increased in ears with > 50% visibility (9.7° vs. 4.3°, *p* = 0.033), demonstrating moderate predictive value (AUROC = 0.689, 95% CI 0.514–0.864) with an optimal threshold of 6.2° (sensitivity 73.4%, specificity 69.2%). Amongst adults, three measurements showed significant associations with visibility: facial recess distance (3.6 mm vs. 2.8 mm, *p* = 0.019), facial nerve location (1.6 mm vs. 1.1 mm, *p* = 0.046), and modified RW niche angle (22.3° vs. 17.7°, *p* = 0.009). The modified RW niche angle was the only significant independent predictor in forward logistic regression analysis (*p* = 0.016), with good diagnostic accuracy (AUROC = 0.805, 95% CI 0.656–0.954) and an optimal threshold of 17.95° (sensitivity 82.2%, specificity 71.4%) (Fig. 5) (Fig. 3e and f).

**Table 3:** Reliability of different variables in black bone sequence between two independent readers

Reliability of different variables in black bone sequence between two independent readers (*n* = 129)			
	*Pediatrics (n=77)*	*Adults (n= 52)*	
	*ICC (95% CI)*	*ICC (95% CI)*	
Facial recess width (mm)	0.540 (0.277–0.708)	0.662 (0.411–0.806)	
Facial recess distance (mm)	0.425 (0.096–0.635)	0.669 (0.424–0.810)	
Facial nerve location (mm)	0.609 (0.384–0.751)	0.527 (0.177–0.729)	
Modified round window niche angle (degree)	0.806 (0.694–0.876)	0.578 (0.265–0.758)	
External auditory canal angle (degree)	0.592 (0.358–0.741)	0.638 (0.369–0.792)

#### Reliability of measurements

There was good reliability for the modified RW niche angle (ICC = 0.806), with moderate reliability for facial recess width (ICC = 0.540), facial nerve location (ICC = 0.609), and EAC angle (ICC = 0.592) for paediatric ears (*n* = 77). There was moderate reliability across all measurements (ICC range 0.527–0.669) (Table 4) including a moderate reliability (ICC = 0.578) for the modified RW niche angle in adult ears (*n* = 52).

**Table 4:** Comparison of different radiological measurements in relation to the operative view of round window niche in the black bone sequence

Comparison of different radiological measurements in relation to the operative view of round window niche in the black bone sequence									
		*> 50% visible RW*		*< 50% visible RW*		*> 50% visible RW*	*< 50% visible RW*		
			*Mean* ± *SD or median (interquartile range)*						
		Pediatrics (*n* = 64)		Pediatrics (*n* = 13)	P-value	Adults (*n* = 45)	Adults (*n* = 7)		P-value
Facial recess width (mm)		4.3 ± 0.8		4.4 ± 0.8	0.437	4.4 ± 0.9	3.9 ± 1.1		0.177
Facial recess distance (mm)		3.3 (2.6, 4.2)		3.1 (2.7, 4.0)	0.946	3.4 ± 2.8	2.8 ± 0.5		0.047*
Facial nerve location (mm)		1.5 ± 0.9		1.4 ± 0.9	0.917	1.5 ± 0.7	0.9 ± 0.6		0.037*
Modified RW niche angle (degree)		22.3 ± 7.0		21.0 ± 6.7	0.537	22.6 ± 5.1	17.9 ± 2.7		0.02*
EAC angle (degree)		9.7 (5.3, 14.8)		4.3 (2.5, 11.6)	0.033*	9.9 ± 6.1	8.2 ± 6.0		0.491

*RW *round window,* EAC *external auditory canal

## Discussion

This study investigated the ability of MRI based landmarks to predict RW visibility at cochlear implantation with a novel BB-MRI sequence. In paediatric patients, the EAC angle was able to distinguish > 50% from < 50% RW visibility, with moderate reliability (ICC = 0.592) and an AUROC of 0.689. Amongst adults, the modified RW niche facial recess distance and facial nerve location, showed significant associations with RW visibility but modified RW niche angle was the sole independent predictor (*P* = 0.016) with excellent performance (AUROC = 0.805). Both the EAC angle (paediatrics) and modified RW niche angle (adults) demonstrated moderate interobserver reliability. In only 5/108 of the initial cohort was it was not possible to identify all landmarks for the BB-MRI measurements, suggesting their potential utility in preoperative planning.

Established CT-based methodologies for predicting RW visibility [1, 3–8, 10, 21, 23] were adapted for BB-MRI. The inconsistent visualization of the chorda tympani nerve on BB-MRI precluded evaluation of measurements such as the Kashio prediction line and direct facial recess width (chorda tympani-to-facial nerve distance). Furthermore, landmarks such as those pertaining to the RW require adaptation for them to be applied to BB-MRI. Unlike previous work focused predominantly on paediatric populations [1, 4–6], our study evaluated both children and adults to enhance applicability. Prior CT studies were also limited by single-reader designs and a requirement for oblique-plane measurements [1, 7, 8, 10, 21].

Kashio et al. studied both EAC angle and facial nerve location in association with RW visibility in a mixed cohort of paediatrics and adults [21]. Our research further evaluates the effects on these measurements on paediatric and adult population separately. The facial nerve location has only significant association in adults but not in paediatrics and this result probably reflects the underlying developmental variation in cochlear orientation during bimodal temporal bone growth [24, 25]. However, the small absolute difference in the measurement in millimeters may limit its clinical utility. The modified RW niche angle, adapted from Fouad et al.‘s CT-based measurement in paediatric patients [5], demonstrated strong discriminative power in adults (AUROC = 0.805) in our study, possibly attributed by its stable anatomic relationship that directly impacts surgical visibility. While we observe a good reliability (ICC = 0.806) of this measurement in paediatric population than in adults, the insignificant association with RW visibility in the paediatric population may again relate to the changing orientation of cochlea basal turn during temporal bone growth rather than unreliable measurement.

The EAC angle is only significant in paediatric population in our study. This result concurs with Kashio et al. [21] and Kang et al. [23], who associated wider angles with improved surgical access. Discrepancies with Rajati et al.‘s null paediatric findings [3] may reflect our larger sample size. However, it showed only modest diagnostic accuracy (AUROC = 0.689) and reliability (ICC = 0.592).

Our findings revealed no significant differences in facial recess width between RW visibility groups for adult or paediatric cohort, aligning with previous CT based studies [5, 21, 23].

Several reasons for the inferior prediction of RW visibility with BB MRI measurements in the paediatric population may be hypothesised. Firstly, there was a substantial median 176-day interval between imaging and surgery - a duration encompassing critical craniofacial growth periods in children. Secondly, some landmarks were more challenging to delineate in the paediatric group, such as the facial nerve which may be obscured by the fatty marrow of an immature diploic mastoid. Thirdly, the bright mucosal line used for EAC demarcation may represent a less consistent landmark in developing temporal bones where the canal’s curvature and orientation undergo progressive changes [25].

The St. Thomas’ Hospital classification system for RW visibility is not universally employed although has been widely applied in the published literature [9, 26–33]. It is important to acknowledge that intraoperative RW visibility is also influenced by dynamic surgical factors which are not captured on pre-operative imaging, such as the angle of the microscope and the degree of dissection, and these may negatively impact on the RW visibility prediction by imaging parameters. However, it should be appreciated that the St. Thomas’ Hospital classification system achieves uniformity by recording round window visibility after optimal exposure, including maximal thinning of the canal wall, maximal skeletonization of the facial nerve posteriorly, and complete removal of the round window niche bony overhang. Thus, the classification system provides a standardized and less subjective framework for assessment by experienced consultant otologists. The strength of our predictive imaging measurements lies in their ability to identify challenging anatomical relationships before any surgical variables are encountered, thus allowing the surgeon to prepare for and adapt the operative approach.

Several limitations should be acknowledged in this study. First, the retrospective design introduces potential selection bias, particularly as 15.7% of cases were excluded due to incomplete surgical records and absence of surgical landmarks on BB-MRI. Secondly, the BB-MRI sequence presents inherent interpretive challenges. This includes the limited differentiation of bone and air interfaces, precluding direct visualization of the RW niche and reducing definition of the posterior wall of the EAC [1]. In addition, the presence of high signal due to the stapedius tendon, chorda tympani and fatty marrow, which confounds identification of the mastoid facial nerve. Thirdly, whilst our study successfully translated CT-derived landmarks to BB-MRI, further refinement using oblique reformatted imaging would better simulates surgical views [34]. Finally, caution is warranted when generalizing these findings to other populations or imaging systems, as results may differ in patients with auricular or inner ear dysplasia or when performing BB-MRI with different systems and sequence parameters. Future prospective studies with standardized imaging protocols across institutions would help validate these preliminary findings.

## Conclusion

The visibility of the RW at posterior tympanotomy may be predicted with pre-operative BB-MRI. The optimal measurements differ between adult and paediatric patients and there is superior performance in adults. The EAC angle and modified RW niche angle can be applied to the paediatric and adult population, respectively for this purpose with moderate reliability.
